# Complications of Intrathecal Baclofen Pump: Prevention and Cure

**DOI:** 10.5402/2012/575168

**Published:** 2012-03-24

**Authors:** Yasser Awaad, Tamer Rizk, Iram Siddiqui, Norbert Roosen, Kelly Mcintosh, G. Michael Waines

**Affiliations:** ^1^Pediatric Neurology and Movement Disorder Program, Oakwood Health System, Dearborn, MI, USA; ^2^Department of Pediatric Neurology, National Neurosciences Institute, King Fahad Medical City, Riyadh 11393, Saudi Arabia; ^3^Department of Neurosurgery, Henry Ford Health Systems, Detroit, MI, USA; ^4^Operating Room Services, Oakwood Health System, Dearborn, MI, USA

## Abstract

Increasingly, spasticity is managed with surgically implanted Intrathecal Baclofen pumps. Intrathecal Baclofen pump revision surgery unrelated to programmable pump end-of-life is not uncommon, requiring special attention during pre-, intra-, and postoperative management. We aimed to identify and describe complications of Intrathecal Baclofen pump as well as to report avoidance and management of complications. *Methods and Materials.* Through 2002–2006, at the department of neurosurgery, Henry Ford and Oakwood Health Systems, Intrathecal Baclofen pumps were implanted in 44 patients: 24 children versus 20 adults; 30 “primary-implant-patients”; 14 “revision-only patients”. We evaluated reasons for revision surgeries and diagnostic workup requirements. *Results.* Eight primary-implant-patients required 14 revisions and 7 of revision-only patients needed 13 procedures. Seven patients with slowly increasing baclofen-resistant spasticity had either (i) unsuspected pump-catheter connector defects, (ii) an X-ray-documented pump-catheter connector defect, (iii) X-ray-demonstrated fractured catheter with intrathecal fragment. Implant infections occurred in 4 cases. Scintigraphy revealed occult CSF leakage *N=1* and intrinsic pump failure *N=1*. *Conclusion.* Intrathecal Baclofen pumps, although very gratifying, have a high, technique-related complication incidence during implant life. Meticulous technique, high clinical suspicion, appropriate workup, and timely surgical management can reduce surgical complications of Intrathecal Baclofen pump implantation.

## 1. Introduction

Intrathecal baclofen (ITB) is becoming a popular modality for management of severe spasticity [1–3]. “Spasticity” is defined as hypertonia in which one or both of the following signs are present (1) resistance to externally imposed movement that increases with increasing speed of stretch and varies with the direction of joint movement, and/or (2) resistance to externally imposed movement rises rapidly above a threshold speed or joint angle [4]. It is a sensorimotor phenomenon related to the integration of the nervous system motor responses to sensory inputs. Although most commonly considered as a velocity-dependent increase to tonic stretch, it is related to hypersensitivity of the reflex arc and changes that occur within the central nervous system (CNS), most notably, the spinal cord. Injury to CNS results in loss of descending inhibition, allowing for the clinical manifestation of abnormal impulses. Muscle activity becomes overactive. This is mediated at several areas of the stretch-reflex pathway. Although spasticity is part of the upper motor neuron syndrome, it is frequently tied to the other presentations of the said syndrome. Contracture, hypertonia, weakness, and movement disorders can all coexist as a result of the upper motor neuron syndrome [5]. Spasticity is a common phenomenon in patients with a wide variety of neurological disorders like cerebral palsy, multiple sclerosis, cardiovascular accidents, strokes, traumatic brain, and spinal cord injury [6, 7]. These patients not only suffer from severe contractures and deformities but also severe pain that incapacitates them.

Appropriate management of spasticity is dependent upon different treatment modalities and requirements. Several oral and parenteral medications have been used to treat spasticity; their mechanisms of action are different and they influence various aspects of the neuromuscular pathophysiology. Benzodiazepines, for example, diazepam exert a medullary and supraspinal inhibitory effect mediated by GABA_A_ receptors [8–13]. Their dosing may need to be progressively increased. Benzodiazepines can lead to habituation; withdrawal symptoms may occur when the medication is abruptly discontinued [8–13]. Side effects such as sedation, memory, and attention deficits are not uncommon. Dantrolene acts directly on the skeletal muscle, without interference with the neurological circuitry; it inhibits release of sequestered calcium from the sarcoplasmic reticulum [10]. Dantrolene can be used also very effectively in combination with diazepam [10]. Adverse effects of dantrolene include general muscle weakness, sedation, and occasionally hepatitis. A third group of antispasticity drugs are *α*_2_ adrenergic agonists, for example, clonidine and tizanidine [11, 12]. They hyperpolarize the motor neuron cell membrane, and they have an antinociceptive effect, which may indirectly reduce muscular hypertonia as well. Side effects include drowsiness, dizziness, nausea, light headedness, vertigo, ataxia, headache, tolerance, and physical dependence. Long-term use is discouraged because of strong abuse potential. The most useful oral medication in the treatment of spasticity is baclofen, a GABA_B_ receptor agonist that causes presynaptic inhibition of mono- and polysynaptic reflex pathways; it also works at the spinal cord level after crossing the blood-brain barrier. An increasing oral dose easily causes side effects including sedation, dizziness, weakness, and dizziness [14, 15].

ITB administration has been introduced, therefore, to maximize Baclofen's effects and minimize its side effects. It has been shown to be useful in the majority of patients to treat severe or intractable spasticity and dystonia leading to a decrease in muscle spasms, pain, and an increase in functional motor activity and control [16–18]. 

We set out to identify and describe complications of ITB pump and to report avoidance and management of complications. Sharing our knowledge regarding patients with ITB is important because as a result of the increasing use of this device, familiarity with the workup of possible implant malfunction is important. There may be diagnostic and technical difficulties. The consequences of the pump malfunction can be detrimental to the patient. Complications of ITB vary from

1. dose related:
  - overdose,
  - withdrawal,
2. mechanical implant dysfunction:
  - tear at metal connector to the pump (within protective silicon covering),
  - inappropriate trimming of catheter,
3. retrieval of old intrathecal catheter fragment through limited hemilaminotomy with durostomy leads to CSF leak with tract formation disturb wound healing, dehiscence, and infection.

Complications can decrease therapeutic effect, thus need to be identified and treated promptly.

## 2. Methods and Materials

A retrospective observational study was conducted aiming to analyze ITB pumps' complications. The patients were followed by single operative and clinical team at the department of neurosurgery at our hospital: Henry Ford and Oakwood Health Systems. After the approval of the hospital internal review board, all ITB pumps inserted at our facility through the period of 2002–2006 were identified (excluding previously performed surgery outside our own personal experience with our patients). A standardised technique for ITB pump implantation was followed “as described later.” Patients who had ITB pump insertion and reasons for insertion were identified and reviewed. We identified those patients who had revision surgery, reasons for revision, and diagnostic workup needed to identify complications as well as type of complications, type of treatment instituted to alleviate complication, and how to identify these complications in the future. Since 2002 ITB pump was implanted in 44 patients:

- 23 F/21 M,
- 24 children (16 F/8 M) versus 20 adults (7 F/13 M),
- 30 “primary-implant-patients” (Table 1) 16 children (10 F/6 M) &14 adults (5 F/9 M),
- 14 “revision-only-patients” (Table 2) 8 children (6 F/2 M) versus 6 adults (2 F/4 M).

Clinical charts and operative and imaging reports were reviewed to evaluate reasons for revision surgery and diagnostic workup requirements.

We analyzed reasons for revision surgeries and diagnostic workup requirements in order to identify these complications early and manage them accordingly.

## 3. Results

**Figure 1:**
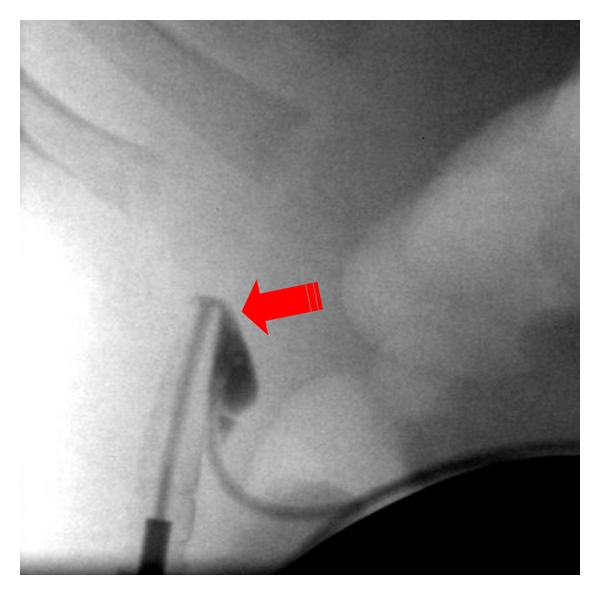
Tear at metal connector to pump within protective silicone covering.

**Figure 2:**
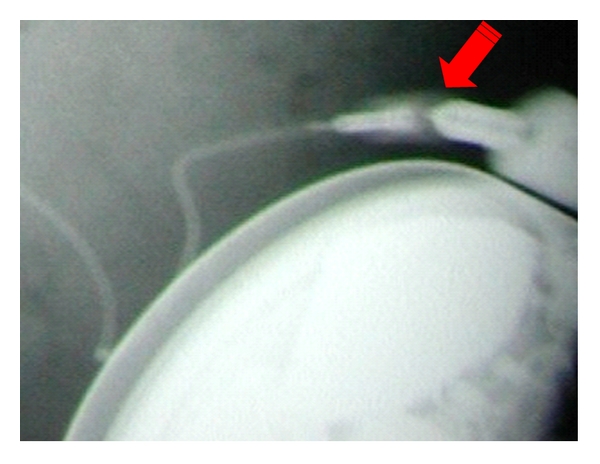
perforating tear at metal connector to pump with protrusion of connector.

Slowly increasing baclofen-resistant spasticity was encountered in seven patients; four of them had unsuspected pump-catheter connector defects with either: subcutaneously dislocated intrathecal catheter *n* = 1, (Figure 1), X-ray-documented pump-catheter connector defect *n* = 1, an eight-years-old boy with posttraumatic spastic quadriplegia and right-sided myoclonic intention tremor. He underwent 18 mL ITB implant on 2002 with good spasticity control then on 2005 left thalamic DBS was done with good tremor control. In 2006, he developed withdrawal symptoms and interventional radiology with pump injection showed tear at metal connector to the pump within protective silicon covering, so revision with trimming of catheter and exchange of the pump to 40 mL pump markedly reduced his Baclofen needs (Figure 2). X-ray demonstrated fractured catheter with intrathecal fragment *n* = 2; the first patient was a 12-year-old boy with spastic quadriplegic CP required laminectomy. His pump was implanted in 2000 with good spasticity control. He underwent multiple revisions in 2001, 2003, and 2004. In 2003, imaging showed adhesive arachnoiditis at the catheter tip. In 2005, he developed mild withdrawal symptoms. Plain X-ray showed fractured catheter with intrathecal fragment. Following retrieval of old IT catheter fragment through limited hemilaminotomy with durotomy, followed by placement of a new IT catheter and replacement of old 10 mL pump with new 40 mL programmable pump, he did not show further CSF accumulation any more. The second patient was managed without laminectomy. Injection studies revealed intrathecal pericatheter arachnoiditis: 53-year-old male with spastic quadriplegic CP with secondary scoliosis. His original ITB was implanted in 2000 with good spasticity control. In 2006, he showed increasing spasticity and scoliosis requiring Baclofen increase. Plain X-ray showed perforating tear at the metal connector to pump with connector protrusion. Revision was done with catheter trimming; exchange of ITB pump to 40 mL pump resulted in marked reduction of his Baclofen needs. The remaining three patients had connector-related dye leakage *n* = 3.

Implant infections occurred in 4 cases (3 with multiple previous revisions). The first one was a three-year-old boy with cerebral palsy, postpump exchange secondary to manufacturer-related mechanical pump failure; his pump was removed, covered with appropriate antibiotics, and then reinsertion of a new pump without further complications followed. The second case was a five-year-old boy with cerebral palsy, postabdominal wound dehiscence, and catheter exposure; antibiotics failed to save the implant, so his pump was removed followed by reinsertion of a new pump without further complications. The third case was an eight-year-old girl with cerebral palsy, postabdominal wound dehiscence, and catheter exposure; her pump was removed; course of appropriate antibiotics was given followed by reinsertion of a new pump without further complications. The fourth case was a twenty-eight-year-old male with cerebral palsy with P. *aeruginosa* in catheter left in situ during previous pump removal (>1 yr) at outside institution treated with appropriate antibiotic. His new implant was not infected

**Figure 3:**
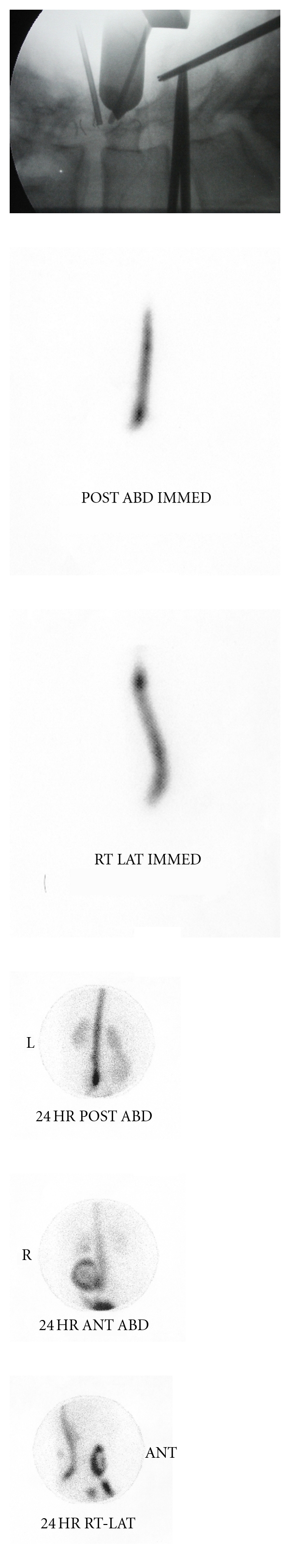
Removal of old catheter with extensive catheter tract obliteration. No specific CSF tract was found. (ABD: Abdominal, ANT: anterior, IMMED: immediately after dye administration, LAT: lateral, POST: posterior, RT: right).

Occult CSF leakage occurred in a 37-year-old male with ITB implanted in 2003 with good spasticity control. In 2004, he developed abdominal CSF collection. Pump injection showed leakage, catheter tear was discovered at pump connector site, and revision surgery was done. He remained to have recurrent CSF collections at abdominal site with negative pump injection studies. Post-LP Scintigraphy revealed lumbar CSF tract. However, no specific CSF tract was found intraoperatively *n* = 1, (see Figure 3); removal of the old intrathecal catheter with extensive catheter tract obliteration and placement of a new IT catheter resulted in no further CSF accumulation anymore.

## 4. Discussion

The difference in effectiveness between oral and intrathecal administration of baclofen is due to a significantly higher drug concentration that can be achieved in the CSF through ITB. In addition, there is a 4 to 1 gradient in drug distribution between the caudal and rostral parts of the spinal cord following ITB, thus providing for a beneficial effect at the spinal level without undesired side effects in the brain [19]. 

Although ITB is highly gratifying, it has predominant technique-related complications during implant life. Meticulous surgical technique, high clinical suspicion, appropriate workup, and timely surgical management are emphasized to reduce surgical ITB complications. Thorough workup is crucial to rule out implant system dysfunction if clinical evaluation is atypical.

Obtaining preoperative imaging, at least a plain X-ray, has been proven to be a safe and effective method of evaluation, although in our centre we have a low threshold to perform pump injection studies with contrast.

In our series, ITB—Revision Surgery for both groups “Primary-Implant-Patient” and “Revision-Only-Patient” Population (*N* = 44). 33 additional revision procedures performed on 22 of 44 patients ITB surgery carry significant morbidity that needs extended care and hospitalization requirements (Tables 1 and 2).

We recommend a standardised implantation technique that can be used as a guide for a safer ITB implants and may help reduce the incidence of complications.

## 5. Standardised ITB Pump Implant Technique

During an informed consent, we elaborated on pros and cons of continuing conservative therapy versus implantation of the ITB. We also acquainted patient of the goals and benefits, possible risks, and complications. Preoperatively, patient's general medical condition was assessed by excluding infection, integrity of the contemplated operative sites, and prophylactic antibiotic within one hour of the incision time. Although the procedure can be performed using local anesthesia and sedation, general anaesthesia was preferred because many of the patients were children.

Incision should be long enough (i.e. 1.5 to 2′′) to expose the wound sufficiently and to minimize potential interaction of the implanted catheter with the skin borders, which are the predominant source of surgical infection.

Then, the lumbar puncture is performed using the 14 G Tuohy needle supplied by the manufacturer in the implant kit. Once the needle tip is in adequate position and CSF flow is achieved, the slightly curved Tuohy needle tip needs to be rotated superiorly in order to guide the catheter superiorly.

The first critical point is when the catheter reached the tip of the Tuohy needle. It is preferable to check fluoroscopically the correct positioning of the catheter at this point: especially important is to ensure that the catheter travels into cranial direction and that it is not looping on itself. The presence of the guide wire and an additional small metallic bead at the catheter tip makes fluoroscopic tracking of the catheter usually relatively easy. Once the initial correct positioning is verified, the catheter can be advanced more, which generally goes without difficulty. The final level of the catheter is determined by the relative spasticity of the upper versus the lower extremities and to what extent spasticity control is targeted for the upper versus the lower extremities.

If the catheter cannot be advanced easily, it is better to leave it at that level instead of forcing it more. If the surgeon decides that the catheter needs to be withdrawn because he noted a looping or the catheter is primarily going into caudal direction, the catheter should never be withdrawn by itself, but rather the Tuohy needle should be withdrawn: otherwise, there is a significant risk to cause a cut in or transection of the catheter.

Once the catheter is in its final position, a purse string stitch is placed very close around the needle penetration site through the fascia. Then the Tuohy needle is withdrawn and subsequently the guide wire. Following removal of the guide wire, the purse string stitch can be tied in such a way that the intrathecal catheter cannot be moved easily anymore through the fascia penetration site. Patency of the catheter should be obvious by the appearance and continuous dripping of CSF from the catheter end. The catheter is now also anchored to the lumbar fascia with a manufacturer supplied anchor, which is attached to the catheter and the fascia with nonresorbable sutures. Take care that this does not result in acute catheter angles that may lead to catheter obliteration. Thus place the catheter in a subcutaneous location around the patient's flank. The catheter end is clamped gently with a rubber shod mosquito to avoid excessive CSF loss.

The abdominal portion of the procedure is usually done at the time of successful lumbar puncture in order to save overall procedural time. The abdominal incision is made and a pocket is created in the subcutaneous adipose layer. The pump pocket is designed such that the pump access ports will not be exactly underneath the incision line. It is useful to have the pump placed on the patient's abdominal fascia because this facilitates secure attachment of the pump and tends to prevent pump hypermobility that can interfere with future pump access for either refill or diagnostic purposes. The pump cavity should be large enough to accommodate the pump. Once the abdominal and the lumbar sites are prepared adequately and the intrathecal catheter is in situ and anchored, a custom-bent tunneler is passed subcutaneously from the abdominal site to the lumbar site. This requires the tunneler to be substantially bent in order to follow the patient's flank around, carefully passing the tunneler through the subcutaneous layer usually without need for counter incisions with guided assistance, tunneling direction towards the lumbar incision. It is important to make sure that enough catheter remains within the lumbar wound to make a small strain relief loop at that site. On the abdominal side, the intrathecal catheter is trimmed as needed to avoid too much looping catheter length but leaving enough material in place to be able to have a loop. At this point, the pump itself is filled with the baclofen solution as per manufacturer procedural guidelines. Patency of the catheter is double-checked when the rubber shod mosquito is removed form the catheter end. The pump-catheter connector is attached to the catheter: this requires gentle manipulation of the catheter to advance it into the connector. The pump is then placed into the pump cavity created earlier, securing the pump to the fascia using the suture loops of the pump. The excess catheter is gently placed underneath the pump prior to securing the device in situ. We feel, using the above-mentioned protocol can reduce the complications, which have been described above.

## 6. Conclusion

The knowledge of the peculiarities of ITB treatment is important, as there is an increased risk for the patients if these implants are not functioning well. The physician who may be called upon to evaluate the ITB pump patient needs to have good understanding of the diagnostic challenges when faced with a possible device malfunction. This also assumes with the familiarity with diagnostic and therapeutic preimplant workup with surgical implant technique and with the postimplant maintenance management.

ITB improves both spasticity and spasms. As a result of reduced spasticity and spasms, patients will be able to sleep better, become more independent with mobility, and their ability to do self-care helps improve urinary function. A decrease in muscle pain and fatigue that accompany spasm may also be seen. Thus effective Baclofen therapy can be delivered using ITB pump, where effects of baclofen are maximised, while its side effects are minimised.

## Figures and Tables

**Table 1:** ITB—implant Surgery patients.

ITB—implant surgery patient population (*N* = 44)				
Age group	Children		Adults	
Gender	M	F	M	F

ITB—indications for implantation				

Cerebral palsy	7	13	8	1
Closed head injury	1	1	1	0
Multiple sclerosis	0	0	1	2
Dystonia	0	1	0	1
Spondylotic cervical myelopathy	0	0	4	0
Intra cranial hemorrhage secondary to hypertension	0	0	0	1
Spasticity	0	0	1	1

**Table 2:** ITB—revision surgery patients.

ITB—revision surgery “primary-implant-patient” population (*N* = 30)				

Age Group	Children		Adults	
Gender	M	F	M	F
Patients requiring revision	4	1	2	1
8 patients required *17* revision procedures:				
(i) *5* revision procedure for 5 patients,				
(ii) *12* revision procedures for 3 patients.				

ITB—revision surgery “revision-only-patient” population (*N* = 14)				

Age Group	Children		Adults	
Gender	M	F	M	F
Patients with previous surgery at outside institutions	2	5	4	3
patients required *16* additional revision procedures.				
(i) 4 patients with previous revisions at outside institutions	1	0	3	0
(a) 4 patients with seven previous revision procedures at				
outside institutions had 6 additional revision procedures in our institution.				
(ii) 10 patients without previous revisions outside	1	5	1	3
(a) 10 patients required *10* revision procedures following the primary implant procedure.				
